# Supplementary material for: Influence of ferromagnetic interlayer exchange coupling on current-induced magnetization switching and Dzyaloshinskii–Moriya interaction in Co/Pt/Co multilayer system
Source: Sci Rep. 2024 Apr 30;14:9938. doi: 10.1038/s41598-024-60492-x (PMC11061319; doi:10.1038/s41598-024-60492-x)
Supplement: Supplementary file 1 — Supplementary Information. [file 41598_2024_60492_MOESM1_ESM.pdf]

Supplementary materials for Influence of Ferromagnetic Interlayer Exchange Coupling on Current-induced Magnetization Switching and Dzyaloshinskii-Moriya Interaction in Co/Pt/Co Multilayer System

1. EXPERIMENTAL DATA

In this section, we provide all Brillouin Light Scattering (BLS) spectra we measured across a wide range of Pt thicknesses (see Fig.S1). The measurements were taken in the Damon-Eschbach mode configuration. Additionally, we show (Fig.S2) the damping determined from the electric measurements (spin-diode (SD) FMR). The damping coefficients (α) were derived from the FWHM of the SD resonance peaks.

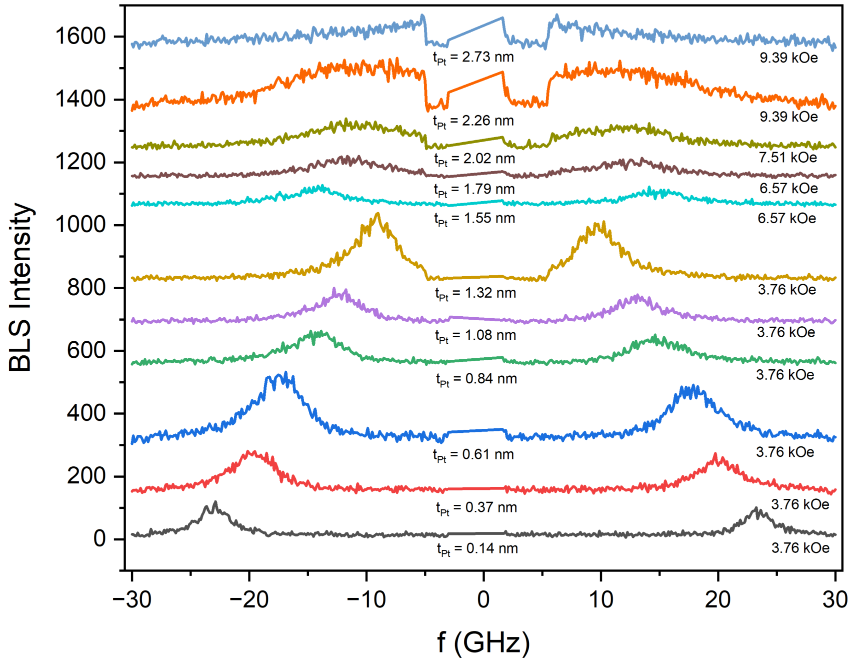

Fig. S1. BLS spectra of Stokes and anti-Stokes peaks for a wide range of Pt thicknesses from regions I-III, where applied magnetic fields range from 3.8 kOe to 9.4 kOe.

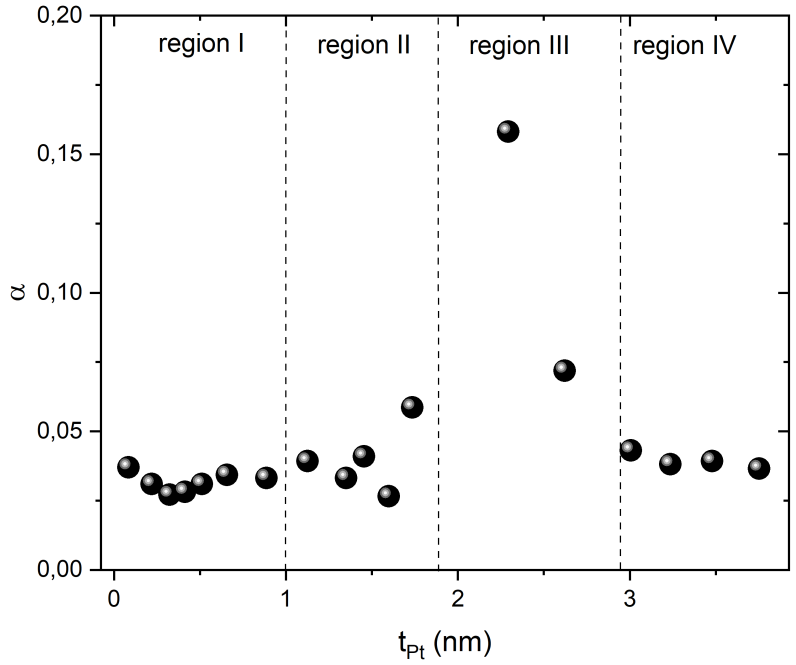

Fig. S2. Damping (α) determined by spin-diode ferromagnetic resonance method (SD-FMR) in wide range of Pt thickness.
